# Multiparametric MRI of Knees in Collegiate Basketball Players: Associations With Morphological Abnormalities and Functional Deficits

**DOI:** 10.1177/23259671231216490

**Published:** 2023-12-14

**Authors:** Cameron Nosrat, Kenneth T. Gao, Rupsa Bhattacharjee, Valentina Pedoia, Matthew F. Koff, Garry E. Gold, Hollis G. Potter, Sharmila Majumdar

**Affiliations:** † Department of Radiology and Biomedical Imaging, University of California, San Francisco, California, USA; ‡ Department of Radiology and Imaging, Hospital for Special Surgery, New York City, New York, USA; § Department of Radiology, Stanford University, Stanford, California, USA; *Investigation performed at the Department of Radiology and Biomedical Imaging, University of California - San Francisco, San Francisco, California, USA*

**Keywords:** articular cartilage, athletic training, basketball, knee, imaging, magnetic resonance

## Abstract

**Background:**

Rates of cartilage degeneration in asymptomatic elite basketball players are significantly higher compared with the general population due to excessive loads on the knee. Compositional quantitative magnetic resonance imaging (qMRI) techniques can identify local biochemical changes of macromolecules observed in cartilage degeneration.

**Purpose/Hypothesis:**

The purpose of this study was to utilize multiparametric qMRI to (1) quantify how T_1ρ_ and T_2_ relaxation times differ based on the presence of anatomic abnormalities and (2) correlate T_1ρ_ and T_2_ with self-reported functional deficits. It was hypothesized that prolonged relaxation times will be associated with knees with MRI-graded abnormalities and knees belonging to basketball players with greater self-reported functional deficits.

**Study Design:**

Cross-sectional study; Level of evidence, 3.

**Methods:**

A total of 75 knees from National Collegiate Athletic Association Division I basketball players (40 female, 35 male) were included in this multicenter study. All players completed the Knee injury and Osteoarthritis Outcome Score (KOOS) and had bilateral knee MRI scans taken. T_1ρ_ and T_2_ were calculated on a voxel-by-voxel basis. The cartilage surfaces were segmented into 6 compartments: lateral femoral condyle, lateral tibia, medial femoral condyle, medial tibia (MT), patella (PAT), and trochlea (TRO). Lesions from the MRI scans were graded for imaging abnormalities, and statistical parametric mapping was performed to study cross-sectional differences based on MRI scan grading of anatomic knee abnormalities. Pearson partial correlations between relaxation times and KOOS subscore values were computed, obtaining *r* value statistical parametric mappings and *P* value clusters.

**Results:**

Knees without patellar tendinosis displayed significantly higher T_1ρ_ in the PAT compared with those with patellar tendinosis (average percentage difference, 10.4%; *P* = .02). Significant prolongation of T_1ρ_ was observed in the MT, TRO, and PAT of knees without compared with those with quadriceps tendinosis (average percentage difference, 12.7%, 13.3%, and 13.4%, respectively; *P* ≤ .05). A weak correlation was found between the KOOS-Symptoms subscale values and T_1ρ_/T_2_.

**Conclusion:**

Certain tissues that bear the brunt of impact developed tendinosis but spared cartilage degeneration. Whereas participants reported minimal functional deficits, their high-impact activities resulted in structural damage that may lead to osteoarthritis after their collegiate careers.

The loadbearing demands of athletes differ by sport and can influence the stress placed on the knee joint, affecting the progression of knee damage and injury.^8,28^ Articular cartilage is a highly specialized connective tissue that not only has a lubricated surface to minimize friction between opposing articulating surfaces but also aids in absorbing large compressive joint loads.^8,28^ The knee is particularly susceptible to cartilage degeneration in athletes involved in high-impact sports, including competitive basketball players, and is thus prone to pain and early-onset osteoarthritis.^8,10,17,26,28^ Imaging modalities, such as magnetic resonance imaging (MRI), are potentially useful to characterize such structural abnormalities.

A previous study of 24 asymptomatic National Collegiate Athletic Association (NCAA) Division I collegiate basketball players found all players to have ≥1 abnormalities on MRI scan.^17^ For example, the prevalence of articular degeneration in asymptomatic basketball players has been estimated to be nearly 50%.^10^ Previous studies have identified several prevalent knee abnormalities on MRI scans in basketball players, including meniscal lesions (20%-54%), fat pad edema (25-41%), patellar tendinopathy (24%-39%), and joint effusion (29%-35%).^10,15,17,26^ Such injuries, and associated alterations in knee joint biomechanics, can influence cartilage degeneration and bone architecture. While these changes may be asymptomatic, they can place high-level athletes at increased risk of irreversible joint degradation and at a higher risk of subsequent need for treatment, including eventual knee replacement. For example, a previous study in former male athletes identified radiographic evidence of osteoarthritis as a risk factor that preceded the development of clinical osteoarthritis and degenerative changes of the lower extremities.^8^ The utilization of quantitative MRI (qMRI) techniques is useful to identify signs of degeneration of articular cartilage before the onset of, often irreversible, structural damage to tissues in high-impact athletes.

Compositional qMRI techniques, including T_1ρ_ and T_2_ relaxation times, are associated with biochemical changes of macromolecules observed in cartilage degeneration, which occur before visible morphological changes that can be captured by imaging.^13,21^ T_1ρ_ is a biomarker sensitive to changes in the extracellular matrix and has been shown to be elevated in populations at risk of, and living with, osteoarthritis^4,20^; T_2_ is inversely associated with organization of collagen fibrils and has been shown to be prolonged in the setting of articular cartilage degeneration.^20^ Previous research has identified associations between cartilage degeneration and prolonged T_1ρ_ and T_2_ relaxation times derived from region of interest (ROI)-based approaches.^4,20^ Due to the limited sensitivity of ROI-based approaches in the segmentation of articular cartilage, more granular and automatic techniques, including statistical-based active shape modeling, texture-based, and atlas-based methods, have been proposed. Voxel-based relaxometry (VBR) has specifically been shown to be a very sensitive, feasible, and accurate tool for assessing biochemical composition of knee cartilage.^18^ VBR allows for the local comparison of cartilage composition on a voxel-by-voxel basis based on a single reference space.^18^ T_1ρ_- and T_2_-derived VBR data permit detection of early biochemical compositional changes and allow more precise identifications of cartilage regions susceptible to degeneration compared with traditional ROI-based approaches.

Previous studies investigating the associations between T_1ρ_ and T_2_ and knee abnormalities have been limited mostly to anterior cruciate ligament (ACL) injuries. Significant elevation of T_1ρ_ and T_2_ have been identified consistently in ACL-injured persons compared with healthy controls.^2,12^ In addition, ROI-based T_1ρ_ and T_2_ values within ACL-injured knees showed significant positive associations with meniscal damage and significant negative associations with functionality.^12^ Other studies have identified elevated T_1ρ_ values in several compartments of posterior cruciate ligament-deficient knees of young athletes and in the medial femoral condyle compartment after ACL reconstruction.^16,19^ However, there exists a scope in understanding how compositional relaxation times could help characterize local associations between cartilage degeneration and the presence of conditions, such as quadriceps tendinosis, patellar tendinosis, and edema.

Patient-reported outcome measures such as Knee injury and Osteoarthritis Outcome Score (KOOS) have been shown to be a reliable and valid instrument to consistently measure knee function in persons with knee injuries.^3^ A previous study found that collegiate basketball players had significantly lower KOOS measures in every domain compared with swimmers,^25^ but similar analyses have yet to correlate local knee changes with these reported functional deficits.

A recent study identified significant prolongation of VBR-derived relaxation times in the medial femoral and tibial compartments and in the posterolateral femur of collegiate basketball players compared with collegiate swimmers,^6^ demonstrating the degenerative changes associated with high-knee impact. Better understanding how such differences in load based on preexisting knee abnormalities contribute to regional cartilage degeneration can inform training, recovery, and treatment of basketball players.

The current study was a hypothesis-driven exploratory analysis investigating previously unanswered associations between multiparametric qMRI, knee abnormalities, functional deficits, and position. The purpose of this study was to (1) elucidate differences in VBR-derived T_1ρ_ and T_2_ relaxation times based on magnetic resonance morphological grading of edema and tendinosis of the quadriceps and patellar tendon and (2) correlate T_1ρ_ and T_2_ relaxation times with self-reported functional deficits (KOOS data). It was hypothesized that persons with MRI-graded injury would display prolonged relaxation times and that prolonged relaxation times would be associated with greater self-reported functional deficits.

## Methods

### Study Demographics

A total of 44 NCAA Division I collegiate basketball players (23 female, 21 male) were recruited for this multicenter study. Players were followed up for 3 seasons, but only data from before their first competitive season were included in this cross-sectional study. Athletes were excluded from the study if they were unlikely to remain in school for the entire study period, had a previous ACL injury from which they had not fully recovered, or had a condition excluding MRI scan (pacemaker, defibrillator, etc). None of the subjects had undergone a meniscectomy or meniscal repair. Procedures were approved by the institutional review boards across 3 participating institutions, and participants from 5 participating schools provided informed written consent. Participant data, including sex, age, height, weight, and site of image acquisition, were deidentified before analysis.

All study participants completed a survey to assess their KOOS before their respective competitive seasons. KOOS is a knee-specific instrument with 5 subscales (Symptoms, Pain, Function in Daily Living [ADL], Function in Sport and Recreation [Sport/Rec], and Knee-Related Quality of Life [QOL]) and is measured on a scale from 0 to 100, with 0 representing extreme functional deficits in the knee and 100 representing no problems.^24^

### MRI Protocol

Bilateral knee imaging was performed using clinical 3-T MRI scanners with an 8- or 18-channel transmit/receive knee coil before the participants’ first competitive season. The protocol included a sagittal 2-dimensional fast spin-echo (FSE) proton density-weighted sequence (field of view [FOV], 16 cm; 512 × 384 matrix; slice thickness, 3 mm; echo train length, 14), a sagittal intermediate-weighted 3-dimensional (3-D) FSE CUBE sequence (repetition time/echo time, 1500/25 ms; FOV, 16 cm; 512 × 512 matrix; slice thickness, 0.7 mm; echo train length, 35; bandwidth, 50 kHz; number of excitations, 0.5), and a 3-D sagittal combined T_1ρ_/T_2_ magnetization-prepared angle modulated portioned k-space spoiled (MAPSS) gradient-echo snapshots research sequence (T_1ρ_ spin-lock time, 0/10/40/80 ms; T_2_ preparation echo time, 0/12.87/25.69/51.39 ms; spin-lock frequency, 500 Hz; FOV, 14 or 16 cm; 256 × 128 matrix; slice thickness, 4 mm).^14^ Multiecho data were fitted on a voxel-by-voxel basis using Levenberg-Marquardt mono-exponential fitting to quantify T_1ρ_ and T_2_ relaxation maps.

### Morphological Characterization

Lesions from MRI scans were graded in a blinded fashion by a board-certified musculoskeletal radiologist (H.G.P.). Cartilage lesions were graded using the modified Noyes score from 0 to 4 (grade 0 indicated no cartilage lesions, grade 1 indicated a signal change, and grades 2 to 4 indicated progressively increasing erosion of the cartilage depth up until the underlying subchondral bone). Images were similarly graded for ligamentous damage (ACL, posterior cruciate ligament, medial collateral ligament, lateral collateral ligament, popliteus, and popliteofibular) and assessed for the presence of osteophytes, sclerosis, synovitis, bone marrow edema, patellar tendon damage (tendinosis), and quadriceps damage (tendinosis). Tendinosis was graded on a scale from 0 to 3 (0, none; 1, <33%; 2, 33% to 66%; 3, 67% to 100%). The MRI grading system used to determine medial and lateral meniscal damage assessed for abnormal meniscal signal intensity^22^ and was graded on a scale from 0 to 3, with 3 corresponding to a definitive meniscal tear. Bone marrow edema was graded on a scale from 0 to 2 (0, none; 1, mild [<1 cm^2^]; 2, severe [>1 cm^2^]).

### Voxel-Based Relaxometry

Images were postprocessed using in-house programs written in MatLab (Version R2021a; MathWorks Inc). First, the VTK CISG registration toolkit was utilized to rigidly register the sagittal MAPSS images in all echoes to the first spin lock time (TSL)/echo time (TE). TSL/TE = 0 of each case followed by the use of Elastix software to allow for nonrigid registration to an atlas.^7,11^ Images were morphed to the same coordinate space, allowing T_1ρ_ and T_2_ values to be calculated per voxel using Levenberg-Marquardt mono-exponential fitting. Semiautomatic edge detection allowed for the cartilage of the atlas to be segmented into 6 compartments: lateral femoral condyle (LFC), lateral tibia (LT), medial femoral condyle (MFC), medial tibia (MT), patella (PAT), and trochlea (TRO).^18^

### Cartilage Segmentation and Thickness Calculation

Due to the potential confounding effect of cartilage thickness on T_1ρ_ and T_2_, additional analyses were conducted to adjust for potential differences in cartilage thickness of each of the 3 masks (patellar, tibial, and femoral compartments) per sagittal slice. A 3-D V-net ensemble was used to automatically segment the cartilages from the CUBE sequence into 5 broader classes: the femoral, tibial, and patellar cartilages; the meniscus and background; and further subdivisions into the MFC, LFC, PAT, TRO, MT, and LT.^1^ Cartilage thickness values for femoral, tibial, and patellar cartilages were then calculated automatically from the segmented masks by using a Euclidean distance transform, followed by skeletonization for each compartment and every sagittal slice.^9^ Values of the distance map were sampled at each skeleton point and were averaged to obtain the mean cartilage thickness for each of the compartments.^9^

### Statistical Analysis

Comparisons of variable outcomes (MRI-graded knee abnormalities, cartilage thickness, KOOS, etc) were conducted utilizing the chi-square test for categorical variables and the analysis of variance for continuous variables. The normality of all outcome variables was analyzed utilizing a Shapiro-Wilks test. In the case of nonnormal variable distributions, continuous variables were analyzed using a Kruskal-Wallis test and categorical variables were analyzed using the Fisher test. Statistical parametric mapping (SPM) was performed to study cross-sectional T_1ρ_ and T_2_ differences between the knees of basketball players with and without abnormalities. T_1ρ_ and T_2_ difference maps were computed for individual voxels from the VBR output, with average percentage differences (APDs) of each cartilage compartment between knees being calculated based on the presence of edema and lesions in the quadriceps and patellar tendon. Summary statistics, including means with standard deviations, were computed for knees with and without morphological defects. The APDs were analyzed in map areas that showed significance. T_1ρ_ and T_2_ Pearson partial correlations with KOOS and MRI grading were computed, obtaining *r* value SPMs and *P* value clusters. Age, sex, body mass index, and site of acquisition were adjusted to control for confounding effects. The significance threshold was set at *P* ≤ .05.

## Results

### Demographic and Morphologic MRI Characteristics of Study Participants

Of the 88 knees included in this study, 13 (14.8%) were excluded from analysis due to scans that were either incomplete, unable to be read, or missing associated sequences, leaving 75 knees for analysis. Of knees with positional designations, 30 (49.2%) belonged to guards, 23 (37.7%) belonged to forwards, and 8 (13.1%) belonged to centers. A majority of the analyzed knees included evidence of patellar tendinosis upon MRI scan grading, with quadriceps tendinosis and bone marrow edema being common as well (Table 1). The KOOS-Sport/Rec and KOOS-QOL subscores were the lowest (ie, worst) in the athletes in our sample (Table 1).

**Table 1:** Characteristics of Knees of Basketball Players (N = 75 Knees)^*a*^

Variable	Value
Age, y	18.69 ± 0.84
Sex	
Male	35 (46.7)
Female	40 (53.3)
BMI, kg/m^2^	23.78 ± 2.56
MRI-graded knee abnormalities	
Bone marrow edema	
0	62 (82.7)
1	11 (14.7)
2	2 (2.7)
Patellar tendinosis	
0	28 (37.3)
1	32 (42.7)
2	14 (18.7)
3	1 (1.3)
Quadriceps tendinosis	
0	46 (61.3)
1	29 (38.7)
KOOS subscale	
Symptoms	87.72 ± 11.61
Pain	90.73 ± 9.55
ADL	96.11 ± 5.26
Sport/Rec	84.32 ± 18.67
QOL	83.26 ± 19.78

a Data are reported as mean ± SD or No. of knees (%). ADL, Function in Daily Living; BMI, body mass index; MRI, magnetic resonance imaging; QOL, Knee-Related Quality of Life; Sport/Rec, Function in Sport and Recreation.

### MRI-Graded Abnormalities

**Figure 1.**
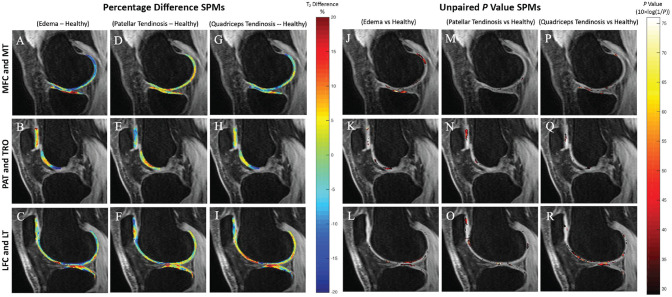
(A-R) Average cross-sectional T_2_ SPMs of all knees overlaid onto registered image. Percentage difference SPMs (A-I) and paired *P* value with a threshold set at *P* ≤ .05 (J-R) show regions of significant differences between knees with MRI-graded knee abnormalities (edema, patellar tendinosis, quadriceps tendinosis) versus healthy knees. In the percentage difference SPMs, red regions indicate higher T_2_ times in knees with an abnormality compared with healthy knees, while blue regions indicate lower T_2_ times. *P* values are represented as 10 × log(1/*P*). Analysis was adjusted for age, sex, BMI, and site of image acquisition. BMI, body mass index; LFC, lateral femoral condyle; LT, lateral tibia; MFC, medial femoral condyle; MRI, magnetic resonance imaging; MT, medial tibia; PAT, patella; SPM, statistical parametric map; TRO, trochlea.

Assessment of the impact of tendinosis or edema on T_2_ relaxation times revealed differences by knee compartment and knee abnormality (Figure 1 and Table 2). Knees with quadriceps tendinosis displayed significantly prolonged T_2_, mostly in the LFC in addition to the LT, compared with knees without quadriceps tendinosis, and LFC T_2_ was correlated with grading of quadriceps tendinosis (percentage of voxels showing significance [PSV] correlated = 13.54%; average *r* = 0.30; *P* = .02).

**Table 2:** VBR Comparison of T_2_ Between Knees With and Without Morphologic Abnormalities^*a*^

Compartment	Bone Marrow Edema			Quadriceps Tendinosis			Patellar Tendinosis		
	PSV, %	APD, %^*b*^	Average *P*	PSV, %	APD, %^*b*^	Average *P*	PSV, %	APD, %^*b*^	Average *P*
MFC	6.30	14.6	.03	5.44	18.1	.03	6.30	19.9	.03
MT	20.36	−17.3	.01	8.40	15.8	.02	6.87	−12.0	.03
LFC	9.08	30.5	.02	13.54	−12.7	.02	4.77	22.7	.03
LT	11.10	−19.4	.01	10.31	−16.3	.02	3.62	−15.1	.03
TRO	5.98	29.7	.02	5.45	−11.8	.04	3.58	−12.4	.02
PAT	3.40	−18.6	.02	4.88	−12.4	.02	19.6	11.1	.03

a PSV, APD, and average *P* values were computed from significantly correlated areas for each abnormality. APD, average percentage difference; BMI, body mass index; LFC, lateral femoral condyle; LT, lateral tibia; MFC, medial femoral condyle; MT, medial tibia; PAT, patella; PSV, percentage of voxels showing significance; TRO, trochlea; VBR, voxel-based relaxometry.

b A negative APD indicates that the T_2_ relaxation times were higher in knees with tendinosis or edema compared with those without. A positive APD indicates that the T_2_ relaxation times were lower in knees with tendinosis or edema compared with those without. Analysis was adjusted for age, sex, BMI, and site of image acquisition.

Knees with patellar tendinosis had a low volume of voxels that displayed prolongation of T_2_ relative to knees without patellar tendinosis (Table 2). Significant T_2_ prolongation was found in the MT and LT compartments of knees with evidence of edema as compared with those without (Table 2). This relationship differed when considering T_1ρ_. Unpaired group analysis of knees with and without quadriceps tendinosis revealed elevated T_1ρ_ in those without tendinosis compared with those with tendinosis, most observed in the MT, TRO, and PAT (Table 3). In addition, knees without patellar tendon tendinosis were found to have elevated T_1ρ_, most commonly in the PAT, compared with those with patellar tendon tendinosis (Table 3). Trends were similar when adjusting our analysis for cartilage thickness in the patellar, tibial, and femoral compartments (Appendix Table A1).

**Table 3:** VBR Comparison of T_1ρ_ Between Basketball Players With and Without Knee Abnormalities^*a*^

Compartment	Bone Marrow Edema			Quadriceps Tendinosis			Patellar Tendinosis		
	PSV, %	APD, %^*b*^	Average *P*	PSV, %	APD, %^*b*^	Average *P*	PSV, %	APD, %^*b*^	Average *P*
MFC	8.20	15.5	.03	2.76	14.2	.03	2.85	−11.9	.03
MT	7.38	21.9	.02	22.14	12.7	.02	4.83	−13.4	.03
LFC	5.15	24.7	.02	8.08	20.02	.02	6.38	−12.8	.03
LT	10.53	−16.8	.02	2.72	−16.3	.03	3.51	−19.4	.03
TRO	8.5	18.88	.03	10.36	13.3	.03	3.34	−11.9	.03
PAT	0.70	−17.2	.03	16.20	13.4	.02	13.06	10.4	.02

a PSV, APD, and average *P* values were computed from significantly correlated areas for each abnormality. APD, average percentage difference; LFC, lateral femoral condyle; LT, lateral tibia; MFC, medial femoral condyle; MT, medial tibia; PAT, patella; PSV, percentage of voxels showing significance; TRO, trochlea; VBR, voxel-based relaxometry.

b A negative APD indicates that the T_*1ρ*_ relaxation times were higher in knees with tendinosis or edema compared with those without. A positive APD indicates that the T_*1ρ*_ relaxation times were lower in knees with tendinosis or edema compared with those without.

### Patient-Reported Outcomes

**Figure 2.**
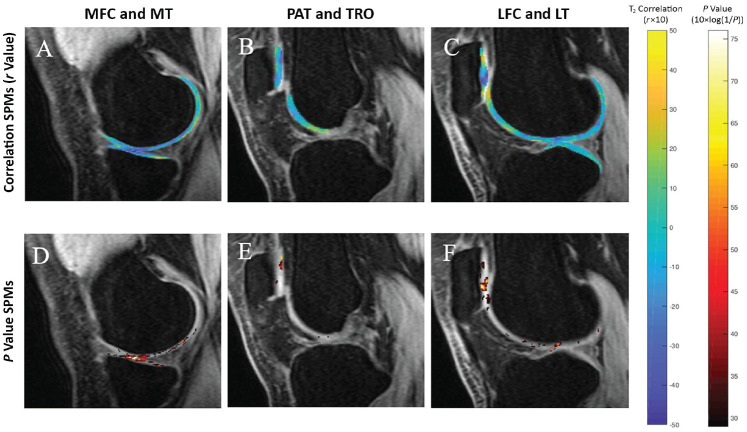
(A-C) Average cross-sectional SPMs of all patients correlating T_2_ values of knees with KOOS-Symptoms values. Subscale values were correlated negatively with T_2_ values in the MFC and MT (A) as well as the PAT (B). A lower KOOS-Symptoms score means symptoms were reported more often. (D-F) *P* value SPMs, reported as 10 × log(1/*P*), identify regions of significance within the correlation SPMs with a significance threshold set at *P* ≤ .05. Analysis was adjusted for age, sex, BMI, and site of image acquisition. BMI, body mass index; KOOS, Knee injury and Osteoarthritis Outcomes Score; LFC, lateral femoral condyle; LT, lateral tibia; MFC, medial femoral condyle; MT, medial tibia; PAT, patella; SPM, statistical parametric map; TRO, trochlea.

The KOOS-Symptoms subscale had the greatest correlation with T_2_ relaxation times of the MT (PSV correlated, 12.98%; average *r* = -0.36; *P* = .02), PAT (PSV correlated, 12.46%; average *r* = -0.36; *P* = .02), and MFC (PSV correlated, 6.30%; average *r* = -0.33; *P* = .02) compartments (Figure 2). The KOOS-Symptoms subscale considers several symptoms, including knee stiffness, swelling, etc, experienced over the past week. The symptom that specifically showed the greatest correlation with T_2_ was knee catching or hanging when moving. This symptom was highly correlated with T_2_ of the MFC (PSV correlated, 26.83%; average *r* = 0.36; *P* = .02), MT (PSV correlated, 18.32%; average *r* = 0.36; *P* = .02), LFC (PSV correlated, 11.38%; average *r* = 0.32; *P* = .02), TRO (PSV correlated, 9.32%; average *r* = 0.32; *P* = .03), and PAT (PSV correlated, 12.54%; average *r* = 0.35; *P* = .02) compartments. Other symptom associations included swelling in the knee and T_2_ of the MT (PSV correlated, 12.21%; average *r* = 0.34; *P* = .02), noise in the knee and T_2_ of the LT (PSV correlated, 8.27%; average *r* = 0.32; *P* = .02) and PAT (PSV correlated, 12.46%; average *r* = 0.34; *P* = .02), and inability to fully straighten knee and T_2_ in the MFC (PSV correlated, 10.35%; average *r* = 0.32; *P* = .02).

Correlations between T_2_ and other KOOS subscales, including Pain, ADL, Sport/Rec, and QOL, displayed weaker correlations. Positive associations were found between T_2_ in the PAT compartment and KOOS-Sport/Rec (PSV correlated, 10.02%; average *r* = 0.32; *P* = .02) as well as KOOS-QOL (PSV correlated, 15.85%; average *r* = 0.33; *P* = .02). In addition, the prolonged relaxation times of the tibial compartment and, more specifically, the MT were most commonly associated with poorer outcomes as reported by total number of KOOS questions (Table 4). The correlations of T_1ρ_ relaxation time with KOOS subscores were similar to the correlations observed in the T_2_ results.

**Table 4:** VBR Correlation Between T_2_ and KOOS Questions

KOOS Question	PSV Correlated, %	Average *r*	*P*
MFC			
Knee catching or hanging when moving	26.83	0.36	.02
Inability to straighten knee fully	10.35	0.32	.02
Difficulty standing	11.91	0.32	.02
Trouble with lack of confidence in knee	12.68	0.35	.02
MT			
Noise in the knee and T_2_ of the LT	8.27	0.32	.02
Swelling in knee	12.21	0.34	.02
Knee catching or hanging when moving	18.32	0.36	.02
Knee pain when twisting	15.52	0.33	.02
Difficulty putting on socks	13.32	0.37	.01
Difficulty getting in and out of bath	18.07	0.35	.02
Difficulty doing heavy domestic duties	13.48	0.35	.02
Difficulty squatting	18.07	0.32	.02
Difficulty twisting or pivoting their knee	14.76	0.35	.02
Trouble with lack of confidence in knee	19.08	0.33	.02
LFC			
Knee catching or hanging when moving	11.38	0.32	.02
LT			
Knee pain at night while in bed	19.48	0.52	.01
Difficult descending stairs	12.68	0.34	.02
Difficulty lying in bed	19.48	0.52	.01
Difficulty getting on and off toilet	11.44	0.41	.01
TRO			
Knee catching or hanging when moving	9.32	0.32	.03
Knee pain while walking	10.01	0.34	.02
PAT			
Clicking/noise in knee	12.46	0.34	.02
Knee catching or hanging when moving	12.54	0.35	.02

KOOS, Knee injury and Osteoarthritis Outcomes Score; LFC, lateral femoral condyle; LT, lateral tibia; MFC, medial femoral condyle; MT, medial tibia; PAT, patella; PSV, percentage of voxels showing significance; TRO, trochlea; VBR, voxel-based relaxometry.

## Discussion

In this study, we utilized multiparametric qMRI to characterize differences in the articular cartilage of knees of collegiate basketball players based on existing morphological defects and self-reported functional deficits. This study locally characterized cartilage degeneration using VBR-derived T_1ρ_ and T_2_ relaxation times based on the presence of tendinosis and knee edema. Group analysis of differences in cartilage degeneration based on MRI-graded knee abnormalities suggested that the presence of knee edema was associated with prolonged relaxation times. This is consistent with previous research, which has identified an association between knee joint effusion volume, cartilage loss, and subsequent progression to osteoarthritis.^23,27^ However, our hypothesis that tendinosis of the quadriceps tendon and patellar tendon would contribute to prolonged relaxation times due to associated degeneration and inflammation was proven incorrect; in fact, patellar tendinosis was associated with significantly lower T_1ρ_ and T_2_ in the patellar cartilage compartment. Moreover, when considering T_1ρ_, specifically with regard to quadriceps tendinosis, we observed significantly shorter relaxation times in the MT, TRO, and PAT compartments.

T_1ρ_ and T_2_ mapping are techniques for quantification of changes in cartilage matrix biochemistry, but each has a different sensitivity to proteoglycan and collagen concentration and organization and thus to compressive and shear stresses. T_1ρ_ is more sensitive to early proteoglycan depletion, while T_2_ mapping is more sensitive to collagen organization and water content. Through attachment of the quadriceps muscles to the patella, the quadriceps tendon helps straighten the knee and extend the leg. The patellar tendon similarly facilitates this movement by attaching the PAT to the tibial tuberosity. A possible explanation for our study findings could be that injury and external load target specific tissues. As a result, tissues that bear the brunt of impact, such as the patellar and quadriceps tendons in this case, develop tendinosis, resulting in lower loads on the patellar articular cartilage, and, in turn, minimize degeneration of the articular cartilage. Alternatively, players with tendinosis might favor their contralateral leg, thus leading to less loading of the cartilage. Thus, athletes who have concomitant tissue injury do not seem to show the impact of cartilage degeneration, at least in the short term.

The KOOS data suggested that knees with noticeable knee symptoms, including weekly swelling, clicking, stiffness, issues straightening and bending, and, most notably, hanging/catching of the knee, likely have localized degeneration of knee cartilage in the MT, PAT, and MFC compartments. It is common for persons with osteoarthritis to experience their knee catching or locking up when moving from the sitting to standing position. In addition, degeneration of the tibial compartment and, more specifically, the medial tibial compartment in our cohort, is most associated with poorer knee health, as suggested by the large number of outcomes associations with T_2_. Other correlations of KOOS outcomes with T_1ρ_ and T_2_, including reported pain, function, and quality of life, were rather weak. Sometimes, the relationships were the inverse of what may be anticipated, as was the case with T_2_ prolongation in the PAT compartment and higher KOOS-Sport/Rec and KOOS-QOL scores. Interestingly, a previous study of patients with patellofemoral pain did not identify any significant associations between T_2_ and clinical characteristics of osteoarthritis such as knee pain and function.^5^ While KOOS subscores other than KOOS-Symptoms were not associated significantly with T_2_, several other associations with individual characteristics were identified. Our results suggest that T_1ρ_ and T_2_ prolongation are not necessarily indicative of degenerative changes, as these are healthy, young collegiate basketball players competing at an elite level, but rather hint of other probable and unexplored reasons such as overuse of their joints. Whereas these players might report feeling unhurt, their strenuous, high-impact activities are unknowingly deteriorating knee health and may lead to more serious and permanent structural changes that might require more invasive surgeries long after their collegiate careers.

### Limitations

There are several limitations to this study to consider. First, the sample size was limited but similar to that of several previous imaging studies in elite basketball players.^10,15,17,26^ Since this study was hypothesis-generating, future studies can build on our preliminary findings and benefit by utilizing a larger cohort, thus better teasing out the relationship between MRI-graded morphological abnormalities, functional deficits, and relaxation times. Second, our analysis was cross-sectional and may not be predictive of the eventual degeneration of the articular cartilage within the knees; a longitudinal follow-up analysis considering differences in cartilage degeneration following a season of play would be useful to better tease out this relationship. It is also important to add that collegiate athletes will likely stress their knees less following their collegiate careers, and it is possible that MRI scan findings and other abnormalities will reverse over time. In addition, we lack information regarding full lower extremity alignment - an important consideration to be included in future studies. Finally, we did not consider an athlete's injury history in their MRI scan grading.

## Conclusion

In summary, our study identified several associations between multiparametric qMRI and characteristics of collegiate basketball players. In addition to contributing to the growing body of knowledge regarding associations between morphologically graded knee abnormalities and cartilage degeneration, this study was the first to identify associations between local cartilage composition, knee abnormalities including tendinosis and bone marrow edema, and self-reported functional deficits.

## Appendix Table A1

**Table:** VBR Comparison of *T*_2_ Between Knees With and Without Abnormalities, Adjusted for Cartilage Thickness^*a*^

Compartment	Bone Marrow Edema			Quadriceps Tendinosis			Patellar Tendinosis		
	PSV, %	APD, %^*b*^	Average *P*	PSV, %	APD, %^*b*^	Average *P*	PSV, %	APD, %^*b*^	Average *P*
MFC	5.78	14.6	.03	4.57	18.7	.03	3.28	−16.9	.02
MT	19.85	−17.0	.02	8.14	15.7	.02	6.62	−12.0	.03
LFC	8.85	31.0	.02	13.31	−13.0	.02	1.08	28.8	.02
LT	12.12	−19.0	.02	11.44	−16.2	.02	3.17	−15.1	.04
TRO	6.10	29.8	.02	5.39	−12.3	.03	3.58	−12.6	.03
PAT	3.57	−18.6	.02	5.14	−12.2	.02	20.56	11.5	.02

a PSV, APD, and average *P* values were computed from significantly correlated areas for each abnormality. BMI, body mass index; LFC, lateral femoral condyle; LT, lateral tibia; MFC, medial femoral condyle; MT, medial tibia; PAT, patella; PSV, percentage of voxels showing significance; TRO, trochlea; VBR, voxel-based relaxometry.

b A negative APD indicates that the T_2_ relaxation times were higher in knees with tendinosis or edema compared with those without. A positive APD indicates that the T_2_ relaxation times were lower in knees with tendinosis or edema compared with those without. Analysis was adjusted for age, sex, BMI, site of image acquisition, and cartilage thickness (tibia, patella, femur).
